# Cognitive-Affective Transdiagnostic Factors Associated With Vulnerability to Alcohol and Prescription Opioid Use in the Context of Pain

**DOI:** 10.35946/arcr.v41.1.08

**Published:** 2021-07-15

**Authors:** Emily L. Zale, Jessica M. Powers, Joseph W. Ditre

**Affiliations:** 1 Department of Psychology, Binghamton University, Binghamton, New York; 2 Department of Psychology, Syracuse University, Syracuse, New York

**Keywords:** alcohol drinking, analgesics, opioids, pain, motivation, alcohol

## Abstract

The use of alcohol and prescription opioids is common among people in pain and poses significant public health burdens. This review identifies factors associated with motivation to use alcohol and prescription opioids in the context of pain. Pain-relevant, cognitive-affective, transdiagnostic vulnerability factors—expectancies/motives, pain catastrophizing, pain-related anxiety, distress intolerance, anxiety sensitivity, and perceived interrelations—were selected from theoretical conceptualizations of pain and substance use. Searches conducted in PubMed, PsycINFO, and Embase returned 25 studies that examined associations between identified variables of interest and the use of alcohol and prescription opioids in the context of pain. Consistent with a larger literature on pain and substance use, the studies included in this review demonstrated that people with chronic pain are motivated to use alcohol and opioids in response to negative affect and hold expectancies/motives for coping with pain. Vulnerabilities that engender difficulty managing aversive internal states (distress intolerance and anxiety sensitivity) and maladaptive responses to pain (pain-related anxiety and pain catastrophizing) also were implicated in motivation for alcohol and opioid use. Although one study found that pain-related anxiety was associated with co-use of alcohol and opioids, no studies examined simultaneous use. Future research directions that can explicate causal associations, identify patterns of alcohol and opioid co-use, clarify the role of pain in cessation processes, and inform treatment development are discussed.

Pain is a complex, near-universal phenomenon, which can be conceptualized as a motivational state that engenders goal-directed action.^1^ Motivational models of substance use highlight the role of expected effects and suggest that individuals become motivated to use substances when such use is perceived as holding greater value than other available objects or events.^2^,^3^ A rapidly growing empirical literature indicates that the use of substances, including alcohol and prescription opioids, may be a risk factor in the onset and progression of painful conditions, and that pain is a proximal determinant of acute substance administration and may serve as a barrier to cessation.^4^–^6^ Accordingly, an evolving reciprocal model suggests that associations between pain and substance use are likely bidirectional in nature, resulting in the maintenance and worsening of both conditions over time.^4^–^6^ A recent critical review highlighted emerging evidence that chronic pain frequently co-occurs with use of alcohol and opioids, and that co-use (i.e., use of both substances within a given timeframe) likely contributes to opioid overdose-related morbidity and mortality and worse substance-related treatment outcomes.^7^ An important next step in this line of research is to identify potentially modifiable cognitive-affective factors that may underlie or exacerbate motivation to use alcohol and prescription opioids in the context of pain. A focus on processes that contribute to the onset, maintenance, or exacerbation of multiple psychiatric disorders (i.e., “transdiagnostic” factors) can further inform novel treatment targets and intervention development.^8^,^9^

The sections that follow begin with a brief overview of alcohol and opioid use, acute and chronic pain, and guiding theoretical frameworks. The results of studies that examined associations between pain, selected transdiagnostic cognitive-affective factors (derived from prominent theoretical conceptualizations of pain–substance use relations), and alcohol/prescription opioid use patterns/trajectories are then reviewed. Finally, the relevant extant literature is discussed with an emphasis on explicating clinical implications and generating recommendations to help guide future research in this emerging domain.

## ALCOHOL AND PRESCRIPTION OPIOID USE

### Prevalence and Impact

Approximately 50% of American adults consume alcohol each month,^10^ and more than 25% endorse hazardous drinking (i.e., patterns of use associated with increased risk for harmful consequences).^11^,^12^ Alcohol is implicated in nearly 100,000 deaths in the United States each year,^13^ is the third leading cause of preventable death,^14^ and has an annual economic impact of more than $250 billion in lost productivity, health care costs, and criminal justice expenses.^15^ Although opioid prescribing has diminished somewhat in the wake of the opioid epidemic, nearly 20% of all Americans received an opioid prescription in 2017.^16^ Nationally representative data further indicate that more than 12 million Americans misuse prescription opioids each year (i.e., use without a prescription or for a reason other than the purpose for which they were prescribed).^17^ In the United States, prescription opioids are responsible for more than 15,000 overdose deaths^18^ and for an economic burden of greater than $78 billion annually.^19^ Although alcohol and prescription opioids have different pharmacokinetic profiles and substance-specific physiological/subjective effects, they may engender overlapping effects in the central and peripheral nervous systems, including activation of neural circuitry involved in pleasure and reward.^20^ Both substances also are implicated in substance use disorders, which are characterized by maladaptive physiological (i.e., tolerance and withdrawal) and behavioral (e.g., impaired control over use behavior, social impairment as a result of use) consequences of use.^21^

### Alcohol and Opioid Co-Use

Although definitions vary in the literature, in the context of alcohol and opioids, co-use may be characterized as concurrent use (i.e., within a given period of time, such as past month or past year) or simultaneous use (i.e., co-ingestion at the same time or in a closely overlapping period of time).^22^–^24^ Despite contraindications for drinking alcohol while using prescription opioids, emerging data suggest that the prevalence of alcohol and opioid co-use is surprisingly high. For example, in samples recruited from primary care clinics, 36% of patients with a prescription for daily use of an opioid reported consuming alcohol in the last 30 days,^25^ and 9% reported drinking to intoxication on up to 5 days in the past month.^26^ Another study of patients on long-term opioid therapy found that 12% reported drinking alcohol within 2 hours of taking their medication.^27^ Results from a community-derived sample further indicated that individuals who endorse prescription opioid misuse also report using alcohol at high rates, with up to 20% admitting to using alcohol and opioids in the same day.^28^ Finally, nationally representative data indicate that individuals in the United States who meet diagnostic criteria for opioid use disorder (OUD) are nearly twice as likely to also meet criteria for alcohol use disorder (AUD), relative to individuals without OUD.^29^ Both alcohol and opioids are central nervous system depressants, and any form of co-use could lead to dangerous, potentially fatal effects (e.g., liver damage, respiratory depression).^30^ Indeed, co-use of alcohol was involved in 15% of deaths attributed to prescription opioid overdose in 2017.^31^ In addition to heightened morbidity and mortality, a recent critical review found that co-occurring OUD and AUD were associated with poorer treatment outcomes for both disorders, with some evidence that alcohol consumption may increase during medication-assisted treatment for OUD.^7^

### Prevalence and Impact of Pain

Although a handful of documented cases indicate a rare congenital inability to perceive pain,^32^ pain is largely thought of as a universal human experience.^33^ Pain is a highly prevalent public health burden that motivates 50% of annual physician visits in the United States,^34^ with chronic pain engendering an annual economic impact of more than $600 billion in health care costs and lost productivity.^35^ A recent update by the International Association for the Study of Pain defines pain as “an unpleasant sensory and emotional experience associated with, or resembling that associated with, actual or potential tissue damage.”^36^ This definition acknowledges that pain is a complex biopsychosocial phenomenon that involves an interplay of sensory-physiological, cognitive-affective, and behavioral processes, and that the experience of pain cannot be reduced to the activity of sensory neurons. As such, pain may persist beyond expected healing time or in the absence of identified tissue damage. The term chronic pain is typically used to describe pain lasting for at least 3 to 6 months,^37^ and is distinguished from cancer-related pain, which differs in etiology and course.^38^ More than 100 million Americans have a chronic noncancer pain diagnosis,^39^ and recent nationally representative data indicate that on most days, nearly 20 million U.S. adults experience pain that interferes with activities of daily living.^40^ The experience of pain commands attention and motivates action to avoid or limit bodily harm, often resulting in avoidance behaviors that can be adaptive in the short term (e.g., to promote healing).^1^ However, long-term cycles of maladaptive cognitive-affective responses that lead to persistent avoidance (e.g., worry that pain will never end) are thought to be a predominant cause of pain-related disability.^41^ Indeed, chronic pain can have significant negative effects on quality of life and emotional well-being, including interference in occupational functioning, recreational activities, relationships, self-care, physical activities, and sleep.^42^

### Co-Occurring Pain and Use of Alcohol and Opioids

The extant literature suggests that pain co-occurs at high rates with both alcohol and opioid use, potentially in a dose-dependent fashion.^6^ With regard to alcohol use, individuals with chronic pain endorse higher rates of hazardous drinking and are up to two times more likely than the general population to meet criteria for AUD.^6^,^39^,^43^ Greater levels of pain severity^44^ and functional interference have been associated with an increased likelihood of engaging in hazardous drinking patterns and meeting diagnostic criteria for AUD,^45^ respectively. Pain also appears to be more common among individuals who report hazardous alcohol use: 43% of people who experience drinking problems (e.g., adverse consequences or life problems as a result of drinking) and 75% of individuals with AUD have been shown to endorse current moderate to severe pain (vs. 18% in the general population).^46^–^48^ The co-occurrence of pain and prescription opioid use is intuitive given that opioids are prescribed for pain relief, though pain is also common among individuals who report use of opioid analgesics without a prescription.^49^–^51^ A review of pain and prescription opioid misuse found that 49% to 96% of patients seeking treatment for prescription opioid misuse reported chronic pain, and that 82% reported pain as their reason for initiating opioid misuse.^52^ This review further observed a positive association between pain severity and opioid misuse, such that even among patients with chronic pain, pain ratings tended to be higher among those who reported prescription opioid misuse or who met criteria for OUD.

## GUIDING THEORETICAL FRAMEWORKS

### Pain Processing and Negative Reinforcement

Pain is an inherently aversive experience that reliably elicits negative affect and motivates escape/avoidance behaviors.^1^,^41^ Consistent with biopsychosocial conceptualizations of pain, the four-stage model of pain processing posits that negative affect mediates behavioral responses to pain.^4^,^53^ More specifically, this model invokes both acute negative affect (e.g., distress) that is elicited from the immediate sensory experience as well as extended pain affect (e.g., depression, anxiety) that manifests in the context of chronic pain and functional impairment. Both forms of pain-related negative affect are considered sufficient to motivate adaptive and/or maladaptive behavioral efforts to cope with pain; substance use has been identified as a commonly employed maladaptive pain coping response;^4^,^54^ and efforts to modulate affect have been implicated in pain and substance use trajectories.^41^,^55^ Negative reinforcement—the process by which behavioral responses that are perceived to alleviate aversive states become more likely or increase in frequency—is a core component of theoretical models of both pain and substance use.^1^,^55^ Researchers have further hypothesized that as attempts to avoid/reduce pain or negative affect via substance use are reinforced, the use of substances as a primary coping strategy may become more entrenched over time.^5^

### Reciprocal Model of Pain and Substance Use

In drawing upon motivation theory, negative reinforcement frameworks, and the four-stage model of pain processing, a leading reciprocal model posits that pain and substance use interrelate in the manner of a positive feedback loop, resulting in more severe pain, greater functional impairment, and the maintenance of addiction.^4^,^5^ Within this model, the substance use–to–pain pathway acknowledges that although substances such as alcohol and opioids can reduce pain in the short term, chronic substance use has been identified as a unique risk factor in the onset and progression of hyperalgesia (i.e., increased sensitivity to painful stimuli) and persistently painful conditions.^56^–^60^ In the pain–to–substance use pathway, which is most germane to the current review, pain is conceptualized as a potent motivator of substance use. Pain severity has consistently been associated with use of multiple substances (e.g., nicotine/tobacco, cannabis, alcohol),^5^ and human experimental research has shown that pain and pain-related negative affect can increase craving and motivate substance use.^61^–^63^ Indeed, the role of pain as a proximal antecedent to substance use is of growing empirical interest, as highlighted by a recently published Catastrophizing, Anxiety, Negative Urgency, and Expectancy (CANUE) model that emphasizes the influence of negative affect in motivation to self-medicate one’s pain with a variety of addictive substances.^64^ Thus, the reciprocal model of pain and substance use predicts that acute pain serves as a proximal determinant of substance use behavior, and that via repeated exposures and reinforcement, relations between pain and substance use may become more robust in the context of chronic or persistent pain.^5^

### Use of Alcohol and Prescription Opioids in the Context of Pain

Given the high degree of co-occurrence and significant individual/societal costs associated with alcohol and prescription opioid use, the goal of this review is to explicate potentially modifiable factors that are associated with motivation to use and co-use alcohol and prescription opioids in the context of pain.^4^,^5^,^64^ The need to examine pain as a determinant of alcohol and prescription opioid use is further supported by evidence that nearly 25% of patients enrolled in both pain and inpatient substance use treatment programs have endorsed using alcohol to cope with pain, with many citing pain as the primary impetus for hazardous drinking and other substance use.^65^,^66^ Similarly, a systematic review of opioid misuse and chronic pain found that approximately 21% to 29% of patients prescribed opioids for chronic pain engage in misuse (i.e., use other than prescribed).^67^ The CANUE model further suggests that people with chronic pain may be most motivated to self-medicate with substances when they also hold maladaptive pain-related cognitions or are otherwise vulnerable to impulsive behavior when distressed.^64^ Indeed, researchers have highlighted several cognitive-affective transdiagnostic constructs (e.g., expectancies, pain-related anxiety, distress intolerance) implicated in the development, maintenance, and exacerbation of bidirectional relations between pain and substance use.^5^,^9^,^64^

## METHOD

### Selection of Relevant Constructs

The constructs of interest in this review were derived from theoretical frameworks of motivation and pain–substance use relations, with a focus on cognitive-affective constructs that are hypothesized mechanisms of the pain–to–substance use pathway and have been implicated as vulnerabilities to multiple psychiatric disorders.^5^,^9^,^64^ Of particular interest were constructs that are hypothesized causal mediators by which the acute pain experience may serve as a proximal determinant of substance administration, or constructs that may function as moderators (e.g., exacerbating or amplifying existing determinants) or serve to make substance use more salient or incentivized in the context of pain. Modifiable cognitive-affective constructs (briefly described below) were selected as the focus because these constructs have the potential to serve as integrated behavioral targets and to better inform future research and intervention development efforts.

### Expectancies, Motives, and Perceived Interrelations

For the current review, expectancies, motives, and perceived interrelations broadly refer to the extent to which people perceive associations between their pain and substance use.^2^,^5^ Whereas expectancies represent beliefs about what will happen as a result of substance use, motives represent the desired results (i.e., self-reported reasons and valued/desired effects) of substance use.^68^ Perceived interrelations further encompass perceptions regarding the co-occurrence and bidirectional effects of pain and substance use.^69^

### Pain-Related Anxiety and Pain Catastrophizing

Pain-related anxiety is the tendency to respond to actual or anticipated pain with anxiety or fear, which may motivate avoidance behaviors.^70^,^71^ Similarly, pain catastrophizing reflects the tendency to interpret actual or anticipated pain in an exaggerated manner.^72^ The contributions of pain-related anxiety and pain catastrophizing to the onset and maintenance of chronic pain are well recognized.^33^ More recently, theoretical models of pain and substance use have included pain-related anxiety and pain catastrophizing as transdiagnostic factors that are also relevant to multiple substance-related outcomes (e.g., craving, heaviness of use, cessation) among people with chronic pain.^5^,^9^,^73^–^75^

### Anxiety Sensitivity

Anxiety sensitivity (defined as fear of the potential negative consequences related to anxiety-related symptoms and sensations)^76^ is another transdiagnostic factor that is likely relevant to pain–substance use reciprocity.^9^ Research has demonstrated independent, positive associations between anxiety sensitivity and heavy/problematic substance use^77^,^78^ and greater pain impairment/persistence.^79^–^81^ There is also evidence that greater anxiety sensitivity may contribute indirectly to the association between pain and poorer outcomes related to substance use and health.^82^

### Distress Intolerance

Distress intolerance also may contribute to pain and substance use reciprocity. Research has consistently demonstrated positive associations between distress intolerance (defined as the perceived inability to tolerate negative emotional and/or other aversive states),^83^ substance addiction, and poorer cessation/treatment outcomes, including drug and alcohol treatment dropout and substance use relapse.^84^,^85^ There is also evidence that levels of distress intolerance may be higher among individuals with co-occurring pain (vs. no pain in the past month)^86^ and that individuals with high distress intolerance are more likely to endorse substance coping motives.^87^–^89^

### Search Strategy and Study Selection

Literature searches were conducted in PubMed, PsycINFO, and Embase using the terms alcohol drinking OR alcohol-related disorders OR analgesics, opioid OR opioid-related disorders; pain; and expectancies OR motives OR perceived interrelations OR negative affect OR pain-related anxiety OR catastrophizing OR anxiety sensitivity OR distress intolerance. All search criteria were limited to human species and peer-reviewed journals published in English before December 2020. Searches yielded 124 unique records after duplicates were removed. Given this review’s focus on the pain–to–substance use pathway, the authors sought to identify studies that examined alcohol/opioid criterion variables in relation to at least one of the selected constructs. They included studies that utilized pain-related predictor variables (e.g., pain intensity) or were conducted among relevant pain populations (e.g., chronic pain, persons living with HIV). Studies conducted among healthy, non–treatment-seeking samples were included only if (a) the sample was necessary to answer a pain-related research question (e.g., laboratory experimental pain studies that require healthy participants), and (b) the study included at least one other variable of interest. Primary reasons for exclusion were (a) it was not a behavioral study of the pain–to–substance use pathway (81 studies) or not a relevant population (32 studies).

## RESULTS

Twenty-five studies were identified for inclusion (see Table 1).

### Pain as a Motivator of Alcohol Use

#### Expectancies, motives, and perceived interrelations

Initial qualitative and cross-sectional evidence suggest that people with chronic pain hold unique cognitions about how pain and alcohol use are related. First, a qualitative study of 10 people living with HIV who had chronic pain and reported heavy drinking (i.e., more than four or five drinks on one occasion or more than seven to 14 drinks per week for women/men) provided evidence that alcohol may be seen by people with chronic pain as a primary means of coping with both pain and pain-related distress.^90^ A theme emerged in which alcohol was perceived by the participants to be a “harmless alternative” to prescription opioids for pain management. Among a sample of patients seeking treatment for AUD (*N* = 128), high-intensity pain ratings (vs. no or low-intensity pain) were associated with a greater number of self-reported drinks per day and higher alcohol craving, and participants with high-intensity pain were more likely to report normalizing motives for drinking (i.e., “to feel normal”).^91^ Finally, researchers recently developed and validated the Expectancies for Alcohol Analgesia measure, which assesses perceived likelihood of pain relief from drinking. In a sample of 273 people who reported chronic pain and current alcohol use, expectancies for analgesia were associated with reporting greater frequency and quantity of alcohol use and identifying coping as a motive for drinking.^92^

#### Negative affect

Consistent with the larger substance use literature and theoretical conceptualizations of negative affect as a primary motivator of substance use behavior,^3^,^5^,^64^ there is experimental and observational evidence that the experience of pain, by eliciting negative affect, is a proximal determinant of alcohol use. Laboratory models of human pain utilize standardized noxious stimuli that attempt to approximate features of clinical pain conditions (e.g., neuropathic pain, musculoskeletal pain), reliably elicit sensory pain and subjective distress, and have successfully been used to investigate causal associations between pain and tobacco smoking.^60^,^61^ A recent laboratory experiment conducted among hazardous drinkers further provides causal evidence that pain increases motivation to drink alcohol.^93^ Specifically, participants randomized to laboratory pain induction (vs. no pain) reported greater negative affect, which in turn was associated with a greater urge and intention to drink. Prospective evidence derived from two multisite clinical trials for AUD (in the United States and the United Kingdom) provides further evidence that negative affect is a determinant of drinking in the context of pain.^94^ Across both samples (*N* = 2,125), pain severity at the end of treatment predicted frequency and quantity of alcohol use at long-term (9- to 12-month) follow-up. Moreover, pain predicted increased negative affect, which mediated the effect of pain on drinking outcomes.

#### Pain-related anxiety

Although several studies have implicated pain-related anxiety in associations between pain and other substances (e.g., tobacco, cannabis),^5^ the search for this review returned only one study that examined associations between pain-related anxiety and alcohol use. In an online survey of 234 adults with chronic pain, pain-related anxiety was positively associated with alcohol-related consequences (e.g., injuries from drinking, blackouts) and impairment in functioning due to alcohol use (i.e., needing a drink in the morning, inability to stop drinking once started, and failure to fulfill obligations due to drinking).^73^ Moderation analyses further revealed that associations between pain-related anxiety and drinking were significant among men, but not women.

#### Pain catastrophizing

Like pain-related anxiety, pain catastrophizing has been widely studied in relation to tobacco smoking and cannabis use.^5^,^64^ However, the authors identified only one study that tested associations between pain catastrophizing and alcohol use outcomes. That study, which tested associations between pain and alcohol consumption and motives among 128 patients seeking AUD treatment, also examined pain catastrophizing as a predictor variable of all outcomes.^91^ Results indicated that pain catastrophizing was associated with greater alcohol craving, AUD symptoms, and normalizing drinking motives, regardless of pain intensity.

### Pain as a Motivator of Prescription Opioid Use

#### Negative affect

Initial evidence suggests that negative affect mediates proximal associations between pain and prescription opioid use, similar to alcohol use. Although the search did not return any experiments that tested causal association between pain and opioid use in humans, prospective studies that utilized repeated assessments provide evidence for the effects of pain on prescription opioid use via negative affect. First, real-time ecological momentary assessment among 34 patients on long-term opioid therapy for chronic pain indicated that, over the 2-week assessment period (2,285 total observations), patients were more likely to report opioid use during occasions of increased pain, and they consumed higher doses when pain was accompanied by increased negative affect.^95^ Similar results were observed in a daily diary study of patients with pain due to sickle cell disease who were prescribed opioids (*N* = 45). Over the 90-day assessment period, greater levels of pain and negative affect were individually associated with use of opioids at higher doses during the same day, although negative affect was not statistically tested as a mediator.^96^

#### Pain-related anxiety

Results from several cross-sectional studies suggest that pain-related anxiety is associated with multiple indices of prescription opioid misuse among people with chronic pain. First, in an online survey of young adults (*N* = 256) who endorsed moderate to severe past-month pain, greater pain-related anxiety was associated with increased likelihood of self-reported addiction to opioids, history of family concern about opioid use, past use of opioid detoxification, and more opioid-related problems.^97^ Similarly, a study of 164 adults with obesity and chronic pain found that pain-related anxiety was associated with opioid misuse.^98^ In a cross-sectional survey of nearly 400 adults with chronic pain, pain-related anxiety was identified as a statistical mediator of associations between pain severity and opioid misuse.^99^ Finally, in a clinical sample of 61 smokers of tobacco cigarettes living with HIV and recruited from an infectious disease clinic, higher levels of pain-related anxiety were associated with current opioid misuse among men, but not women.^100^

#### Pain catastrophizing

Several studies conducted among treatment-seeking chronic pain samples consistently demonstrated positive associations between pain catastrophizing and prescription opioid use. First, among a sample of 51 patients with chronic pain, two facets of pain catastrophizing were positively associated with higher scores on a measure of risk for opioid misuse.^101^ Specifically, rumination (i.e., the tendency to have difficulty disengaging from pain-related cognitions) and magnification (i.e., the tendency to magnify perceptions of threat) were each individually associated with risk of opioid misuse. There was also evidence that pain catastrophizing was associated with more frequent cravings for opioids, regardless of pain intensity.^102^ Similarly, a daily diary study revealed that people with chronic pain maintained on opioid therapy used higher dosages of their prescription opioids on days in which self-reported catastrophizing was higher, even when pain was low.^96^ In addition, two cross-sectional studies conducted among patients maintained on opioid therapy for chronic pain demonstrated evidence of negative affect as a statistical mediator of associations between pain catastrophizing and opioid misuse.^103^,^104^

#### Anxiety sensitivity

Among an online sample of 429 adults who self-reported moderate to severe chronic pain and prescription opioid use, anxiety sensitivity mediated associations between greater pain intensity and opioid misuse and OUD symptoms^105^,^106^ and was associated with greater likelihood of endorsing use of opioid medications “to get high.”^78^ In the same sample, models of indirect effects indicated that negative affect was associated with opioid misuse through anxiety sensitivity.^107^ Interestingly, when the model was run in reverse, equal statistical support was observed for an indirect effect of anxiety sensitivity via negative affect, suggesting that prescription opioid misuse may occur in the context of a complex interplay between negative affect and anxiety sensitivity. Finally, data derived from an online sample of nearly 300 adults who reported chronic low back pain indicated that anxiety sensitivity may have an indirect effect on risk of opioid misuse through greater coping and pain management motives for prescription opioid use.^108^

#### Distress intolerance

Distress intolerance previously has been associated with tobacco and cannabis use among people with chronic pain and has been implicated in heavy drinking and alcohol-related problems in healthy populations.^5^,^109^ However, only one cross-sectional study that investigated associations between distress intolerance and prescription opioid use was identified. Among a sample of 39 patients at a pain management clinic who were prescribed opioids, greater levels of distress intolerance were associated with greater scores on a measure of opioid misuse risk, even after statistical analyses controlled for pain intensity.^110^

### Pain as a Motivator of Alcohol and Opioid Co-Use

Although co-use of alcohol and opioids has potentially dire health consequences, this review identified only one study that directly examined alcohol and prescription opioid co-use in the context of chronic pain. In an online sample of 1,812 adults with chronic low back pain, 12% endorsed use of both alcohol and prescription opioids (co-use) and 3% met cut-offs for both hazardous drinking and opioid misuse in the past month (i.e., concurrent use).^111^ Pain-related anxiety was individually associated with hazardous alcohol use, opioid misuse, and likelihood of alcohol and opioid co-use. Moreover, every 1-point increase in pain-related anxiety was associated with a 4% increase in likelihood of concurrent hazardous drinking and opioid misuse. Another recently published study, which examined polysubstance use (defined as use of more than one substance in the month before treatment) among a subsample of 236 people receiving inpatient treatment for AUD or OUD who reported chronic pain, showed that the two most commonly reported substances were alcohol and prescription opioids.^112^ Separate statistical models further indicated that pain-related interference with functioning was associated with a greater number of substances used among men (but not women) and among people with AUD (but not people with OUD). No associations were observed between pain catastrophizing and number of substances used.

## DISCUSSION

Prior research has demonstrated that pain motivates substance use and may contribute to the maintenance of addiction.^5^,^6^,^64^ The purpose of the current review was to examine modifiable cognitive-affective factors that may be associated with motivation to use alcohol and prescription opioids in the context of pain. These constructs include key mechanisms in motivational models of both substance use and pain (negative affect, expectancies/motives)^2^,^113^ and factors in the reciprocal model of pain and substance abuse (pain-related anxiety, pain catastrophizing, distress intolerance, and anxiety sensitivity) that may increase vulnerability to both conditions.^5^,^9^,^64^ Consistent with a reciprocal model of pain and substance use, this review provides evidence that pain is a proximal determinant of alcohol use^114^ and opioid use,^95^ even among individuals without chronic pain.^114^ The review further observed consistent evidence that people with chronic pain are motivated to use alcohol and prescription opioids in response to negative affect^94^,^95^ and maladaptive pain-related cognitions (e.g., catastrophic thinking).^91^,^100^ Finally, this review found initial evidence suggesting that difficulty managing aversive internal states is associated with risk for opioid misuse,^110^ and that people with chronic pain hold unique motives and perceptions about how their pain and drinking are interrelated.^90^

Motivational models of both pain and substance use highlight the role of negative affect as a primary determinant of escape/avoidance behaviors, and negative reinforcement is likely a key mechanism by which pain motivates and ultimately maintains substance use.^5^,^64^ The current review provides some support for this perspective, with experimental and real-time evidence from three studies indicating that negative affect mediates associations between the experience of pain and acute bouts of alcohol and prescription opioid use.^93^,^95^,^96^ The authors also reviewed three studies that provided cross-sectional evidence of covariation between negative affect and transdiagnostic vulnerability factors, such that negative affect was a statistical mediator of associations between opioid misuse and both pain catastrophizing^103^,^104^ and anxiety sensitivity.^107^ Initial evidence from one study further suggests that difficulty with tolerating negative affect (i.e., distress intolerance) is associated with opioid misuse.^110^ Taken together, these findings lend support to the notion that people may experience greater motivation to use alcohol and prescription opioids during heightened states of acute and extended pain affect, and that such effects may be amplified in the context of transdiagnostic vulnerability factors that exacerbate (e.g., pain catastrophizing) or diminish capacity for coping with (e.g., distress intolerance) negative affect.

Pain-related anxiety and pain catastrophizing are both thought to motivate maladaptive attempts to avoid or alleviate pain.^115^ Consistent with evidence that both prescription opioids and alcohol have acute analgesic effects, the current review provides initial evidence that people who experience chronic pain may view drinking alcohol as a viable approach to pain management^90^ or hold expectancies for pain relief from drinking.^92^ The review also observed consistent evidence across several studies that pain-related anxiety and pain catastrophizing are associated with alcohol and opioid use among people with chronic pain.^73^,^96^,^102^,^111^ Maladaptive cognitive-affective responses to pain may activate escape/avoidance processes, leading to use of alcohol and/or opioids. These observations are in line with conceptual models of pain and substance use and further support consideration of pain-related cognitions as potentially key transdiagnostic vulnerability factors for alcohol and opioid use.^5^,^9^,^64^

Although both alcohol and prescription opioids present health risks when used individually, co-use is associated with increased morbidity and mortality and presents a significant public health threat.^7^ Despite the dangers of concurrent use of alcohol and opioids, the authors found only one study that was designed to examine co-use of these substances in the context of pain.^111^ Consistent with findings from studies of either substance alone, pain-related anxiety was associated with greater likelihood of misuse of both substances concurrently in an online sample of adults with chronic pain. One potential explanation for this finding is that people with higher levels of pain-related anxiety may view concurrent use as a way to extend or supplement analgesic effects of both substances.^116^ Although there is reason to suspect that alcohol-opioid co-use also could be seen as a more potent means of escaping/avoiding negative affect (vs. use of either substance alone), this hypothesis is yet to be tested.

### Limitations and Future Directions

Studies included in the current review consistently yielded evidence suggesting that negative affect and other maladaptive cognitive-affective responses to pain and distress may cause alcohol and prescription opioids to take on greater salience in the context of pain. As shown in Table 1, this review identified one experimental study and three prospective studies that lend support regarding temporal precedence; however, the majority of reviewed studies were cross-sectional in nature and thus preclude causal interpretations. Future research would benefit from employing experimental and prospective designs to identify causal relationships and monitor covariation between pain-relevant cognitive-affective constructs and the use or co-use of alcohol and/or opioids over time. For example, prospective studies may test whether maladaptive responses to pain predict escalation of alcohol and/or opioid use or the development of AUD and/or OUD. Ecological momentary assessment provides a promising avenue for assessment of alcohol and opioid co-use in real time and should be considered as a means of better understanding simultaneous use. Indeed, despite the dangers of being under the influence of alcohol and opioids at the same time, the current review did not identify any studies that have investigated motivation for simultaneous use in relation to this review’s constructs of interest. Although dichotomous co-use status (yes/no) provides utility at this early stage of hypothesis testing, co-use should be assessed in greater detail (e.g., frequency, quantity, experience of negative consequences, and temporal proximity of alcohol and prescription opioid ingestion). Particular attention should be paid to identifying patterns of heavy drinking (e.g., frequency, quantity) and prescription opioid use (e.g., high dose, prolonged release, and once-daily formulation) that may increase risk of overdose or other harmful effects.^31^,^117^ Research in this area should include a focus on both overlapping and distinct pharmacologic effects of alcohol and prescription opioids.

Future research also is needed to better understand motivation to use alcohol and prescription opioids in the context of other comorbidities. First, co-use of alcohol and opioids with other substances (e.g., nicotine, cannabis)—particularly those that increase risk for medical consequences and public health burden—should be examined. For example, 22% of opioid-related overdose deaths involve co-use of alcohol, opioids, and benzodiazepines.^118^ Up to 23% of chronic pain patients prescribed opioids also hold a concurrent prescription for a benzodiazepine,^119^ and risk of opioid-related overdose death among this group is 10 times greater than among those who hold an opioid prescription alone.^120^ Anxiolytic properties of benzodiazepines may further encourage use as a method to escape/avoid pain and negative affect. Similar considerations regarding temporal precedence and the need for comprehensive assessment of dynamic substance use patterns should be applied to the study of polysubstance use in the context of pain. This review’s transdiagnostic approach, which focuses on vulnerability factors implicated in a range of psychiatric conditions (e.g., depression, post-traumatic stress disorder, anxiety, personality disorders),^121^–^124^ also highlights the complexity and interrelatedness of pain, substance use, and psychiatric comorbidities. Future research should seek to identify additional vulnerability factors that may contribute to or exacerbate relations between pain and use or co-use of alcohol or prescription opioids. For example, separate literatures have shown bidirectional relationships between sleep disturbances (e.g., difficulty falling or staying asleep) and both substance use^125^ and pain;^126^ researchers should consider a range of behavioral and psychiatric comorbidities when studying alcohol and prescription opioid use in the context of pain. Finally, the majority of included studies focused on a single construct of interest, and additional studies should be conducted to examine interrelations and unique contributions of the constructs examined in this review. Indeed, several studies examined negative affect as a statistical mediator of associations between cognitive-affective factors (i.e., anxiety sensitivity, catastrophizing) and opioid use,^103^,^104^,^107^ and only one study tested associations between a cognitive-affective variable and self-reported motives for opioid use.^108^ Future research is needed to disentangle likely complex and bidirectional associations between this review’s variables of interest.

Given the central role of motives and expectancies in motivational models of substance use,^3^ the authors were surprised to find a paucity of studies that examined expectancies and motives using validated measures (e.g., the Alcohol Expectancy Questionnaire^127^). Albeit limited, data from this review are in line with the larger literature suggesting that people with chronic pain hold substance-related outcome expectancies for pain relief and coping.^5^ Recent validation of the Expectancies for Alcohol Analgesia scale provides evidence that people with chronic pain can reliably self-report expectancies for pain relief from alcohol and that perceived likelihood of analgesic effects may motivate greater frequency and/or quantity of drinking.^92^ In addition to coping, future research is needed to identify other types of motives and expectancies that may motivate substance use in the context of pain. For example, chronic pain often results in decreased social functioning, and researchers have hypothesized that social drinking motives may be particularly salient as a means of reducing pain-related interference in social functioning.^6^ Similarly, as chronic pain interferes with occupational, social, and recreational functioning, people who have high levels of pain-related disability often lose access to other positive reinforcers.^4^ A key component of incentive motivation models involves weighing the incentive value of substance use against other incentives available in the environment.^2^,^113^ For people with chronic pain, expectancies regarding the positive reinforcing effects (e.g., to feel good or high) may become particularly salient. Future research should assess a range of expectancies and motives (including and beyond coping with pain and direct pain reduction) for alcohol, opioids, and co-use of both substances among people with chronic pain. Future research also would benefit from examining the role of positive affect and positive reinforcement processes in bidirectional alcohol and opioid use processes.

Although only a few included studies examined sex/gender differences, this review did observe initial evidence that pain and pain-related constructs may hold greater motivational salience for alcohol and prescription opioid use among men, relative to women. Future research should investigate reasons why men may be more motivated to use substances in the context of pain. For example, men and women likely experience varying pharmacologic effects of alcohol and opioids as a function of biological sex,^128^ and it may be that men derive greater analgesia from use or co-use of alcohol and prescription opioids.^129^ Considering gender as a biopsychosocial construct,^130^ there is reason to suspect that men and women experience pain and substance use differently. For example, masculine norms may influence which pain-related behaviors are most common or accessible to men,^131^ which is consistent with a larger literature indicating that men are more likely than women to cope with pain and anxiety by using externalizing strategies.^132^,^133^ Thus, future studies that consider pharmacokinetic properties of prescription opioids and alcohol should examine sex as a biological variable, and research that investigates psychosocial and behavioral constructs should explore gender differences. Although the findings presented in the current review may have been drawn from cisgender samples, future research should test associations between pain and substance use among transgender and gender minority populations.^134^,^135^ Additionally, despite documented racial/ethnic disparities in the prevalence, treatment, and outcomes of pain-related conditions,^136^,^137^ this review identified only one study that examined the current constructs of interest among racial/ethnic minorities.^97^ A recent review of racial and ethnic disparities in chronic pain treatment found that Black patients maintained on long-term opioid medication were more closely monitored for misuse, despite higher rates of opioid misuse and opioid-related overdose deaths observed among Whites.^138^ Furthermore, racial and ethnic minorities, including Native Americans, Blacks, and Hispanics, are disproportionately impacted by drinking, including greater alcohol-related problems and reduced access to treatment, compared to Whites.^139^ Initial evidence derived from the tobacco literature suggests there may be important racial/ethnic differences in associations between cognitive-affective constructs, pain experience, and substance use.^140^ Future research would benefit from examining disparities as a function of social/cultural characteristics, racial/ethnic discrimination, and economic disadvantage, among others.

Finally, this review identified studies that predominantly focused on motivational processes in the context of ongoing substance use. Additional research is needed to better understand whether cognitive-affective and transdiagnostic vulnerability factors are also associated with motivational processes in the context of substance-related cessation, reduction, and abstinence. For example, several constructs (e.g., pain-related anxiety) previously were associated with relapse to tobacco smoking among people with chronic pain.^75^ Although this review identified one study that examined long-term outcomes after participation in treatment for AUD, the authors are not aware of any work that has directly tested these constructs in relation to lapse/relapse trajectories for alcohol and/or prescription opioid use. Consistent with a phase-based approach to treating substance use,^141^ research should focus on the full spectrum of substance use outcomes rather than on long-term cessation rates alone. Future research also should seek to identify the extent to which expectancies for pain relief and cognitive-affective responses to pain may be related to motivation to quit or reduce use or co-use of alcohol and prescription opioids, or whether such factors influence differential acceptance of referral to alcohol treatment or to programs for tapering prescription opioid use.

### Clinical Implications

Although based on a relatively nascent empirical literature, the current review highlights the importance of assessing pain when treating alcohol and prescription opioid use. Integrated treatments may be especially useful for addressing co-occurring pain and substance use because, relative to traditional approaches (e.g., distinct treatments for individual disorders delivered sequentially), they can be more efficient and cost-effective and can focus on both conditions as treatment targets.^142^ The currently reviewed cognitive-affective constructs represent intuitive targets for integrated treatments because of their likely transdiagnostic role in both substance- and pain-related processes across multiple disorders. Indeed, substance use interventions that address transdiagnostic vulnerabilities (e.g., anxiety sensitivity) are well underway,^143^,^144^ and this work should be extended to the domain of alcohol and opioid use or co-use. Several evidence-based techniques have been used to address maladaptive appraisals of pain and negative affect, including cognitive restructuring (i.e., development of balanced, adaptive thought patterns),^145^ graded exposure to pain and distress-eliciting stimuli (i.e., to reduce escape/avoidance behaviors),^146^,^147^ and coping skills training (e.g., skills to tolerate distress and pain without substance use).^148^–^150^ In addition, integrated behavioral interventions may be utilized in concert with pharmacotherapy, and several pharmacotherapies that are commonly used for pain management (e.g., gabapentin, bupropion) are under investigation for their utility in the treatment of alcohol use and misuse.^151^–^153^ Future work should consider whether there may be synergistic benefit to including pharmacotherapy in integrated interventions for pain and substance use.

Motivational enhancement interventions that target treatment engagement (e.g., willingness to accept a referral for opioid tapering) and motivation to reduce or abstain from alcohol and opioid use also are needed. Although interventions to increase awareness of opioid overdose risk behaviors have been developed,^154^,^155^ the authors are not aware of any treatments that address alcohol and opioid co-use in the context of pain. An integrated treatment for alcohol and opioid use could focus on increasing knowledge regarding adverse interrelations between pain, alcohol, and opioids; increasing motivation and intention to reduce hazardous alcohol use and misuse of opioid medications; and reducing intentions to co-use alcohol and prescription opioid medications. Consistent with a motivational enhancement approach, an important intervention component would be to make an explicit link between continued alcohol/opioid use and poorer pain outcomes, and to highlight pain-related benefits of cessation (i.e., clinically meaningful improvements in pain and interference have been documented after reducing hazardous drinking^6^,^156^ and opioid tapering^157^).^158^ Indeed, recent studies derived from the tobacco literature suggest that chronic pain patients may be motivated to reduce their substance use once they perceive a discrepancy between continued substance use and desired pain outcomes.^159^,^160^

Finally, personalized feedback interventions (PFIs) are a subset of motivational interventions that show greater promise for treating co-occurring pain and substance use, and can be delivered via scalable treatment modalities (e.g., computer-delivered interventions, smartphone applications).^161^ PFIs motivate behavior change via psychoeducation and presentation of feedback about personal behavior (e.g., risk severity) in normative comparison to others (e.g., from relevant sociodemographic groups).^162^,^163^ Parallel lines of inquiry indicate that PFIs decrease maladaptive cognitive-affective and behavioral responses to pain,^164^,^165^ and that they reduce hazardous drinking as well as progression to and maintenance of AUD.^166^,^167^ Two recent studies from the tobacco literature indicated that a brief, single-session computerized PFI is sufficient to increase knowledge of interrelationships between pain, opioid use, and smoking^168^ and motivation/intention to quit smoking.^159^ Computerized or smartphone-based PFIs can be integrated efficiently with self-monitoring and momentary assessment tools to provide immediate or same-day personalized feedback or just-in-time adaptive interventions (i.e., delivery of intervention content that has been adapted based on time-varying factors, including the state of vulnerability and receptivity to support), and can be used to increase treatment engagement.^164^,^169^,^170^ These results provide support regarding the potential of integrated PFI interventions for pain, alcohol, and opioids.

## CONCLUSIONS

Chronic pain and use of alcohol and prescription opioids co-occur frequently, and pain is a potent motivator of alcohol and opioid use. People with chronic pain may be motivated to use alcohol and opioids in response to negative affect or in response to expectancies/motives for pain coping. Transdiagnostic vulnerabilities for maladaptive responses to pain (pain-related anxiety, pain catastrophizing) and difficulty managing or tolerating aversive states and negative affect (distress intolerance, anxiety sensitivity) also may motivate alcohol or opioid use in the context of pain. Future research should examine the role of transdiagnostic factors in motivating patterns of alcohol or opioid use or co-use over time (e.g., escalations in frequency/quantity of use, lapse/relapse trajectories). Integrated interventions for alcohol and prescription opioid use that address pain-relevant, cognitive-affective processes (e.g., motivation for escape/avoidance of pain or negative affect) also should be developed and tested.

## Figures and Tables

**Table 1:** References Identified in Literature Search (*N* = 25), by Variable of Interest

Reference	Author	Year	Design	Outcome(s)
**Expectancies, Motives, and Perceived Interrelations**				
90	Palfai et al.	2019	Cross-sectional	Alcohol Use
91	Nieto et al.	2020	Cross-sectional	Alcohol Use
92	LaRowe, Maisto, & Ditre	2021	Cross-sectional	Alcohol Use
**Negative Affect**				
93	Moskal et al.	2018	Experimental	Alcohol Use
94	Witkiewitz et al.	2015	Longitudinal	Alcohol Use
95	Carpenter et al.	2019	EMA*	Opioid Use
96	Finan et al.	2018	Daily Diary	Opioid Use
**Pain-Related Anxiety**				
73	Zale et al.	2019	Cross-sectional	Alcohol Use
97	Rogers et al.	2018	Cross-sectional	Opioid Use
98	Rogers et al.	2020	Cross-sectional	Opioid Use
99	Rogers et al.	2020	Cross-sectional	Opioid Use
100	LaRowe et al.	2018	Cross-sectional	Opioid Use
111	LaRowe et al.	2020	Cross-sectional	Alcohol Opioid Co-Use
**Pain Catastrophizing**				
91	Nieto et al.	2020	Cross-sectional	Alcohol Use
101	Lee et al.	2020	Cross-sectional	Opioid Use
96	Finan et al.	2018	EMA*	Opioid Use
102	Martel et al.	2014	Cross-sectional	Opioid Use
103	Arteta et al.	2016	Cross-sectional	Opioid Use
104	Martel et al.	2013	Cross-sectional	Opioid Use
112	Votaw et al.	2020	Cross-sectional	Alcohol Use, Opioid Use
**Anxiety Sensitivity**				
105 †	Rogers et al.	2019	Cross-sectional	Opioid Use
106 †	Smit et al.	2020	Cross-sectional	Opioid Use
78 †	Rogers et al.	2019	Cross-sectional	Opioid Use
107 †	Rogers et al.	2020	Cross-sectional	Opioid Use
108	Rogers et al.	2020	Cross-sectional	Opioid Use
**Distress Intolerance**				
110	McHugh et al.	2014	Cross-sectional	Opioid Use

* EMA, ecological momentary assessment.

† Studies were drawn from the same sample.
